# Stigma

**DOI:** 10.4269/ajtmh.21-1033

**Published:** 2022-01-10

**Authors:** Sebin George Abraham

**Affiliations:** Christian Fellowship Hospital, Oddanchatram, Dindigul, Tamil Nadu, India

It was a bright Wednesday morning when our protagonist—whom I shall henceforth refer to as Sharika/Ms. S—walked into our outpatient department. She came along with her mother, who looked tired but resolute, ready to take on the world irrespective of what it threw at her. Sharika was 10 years old but weighed little more than a 6-year-old and appeared timid, almost hiding behind her mother’s thin frame. She did warm up to me after a while and started answering queries about her weight loss and frequent urination, but appeared alarmed when I mentioned the need for blood tests and hospitalization to her mother. She began to plead with me, requesting to avoid blood tests. Despite my attempt at cajoling, she came back teary eyed after a simple blood sugar test that required only a skin prick. As expected, her blood sugar level was close to 300 mg/dL, which confirmed my suspicion that Sharika’s symptoms were caused by type 1 diabetes mellitus.

I began to explain the disease to her mother cautiously. I talked briefly about the implications of being diagnosed with type 1 diabetes mellitus and described the need for regular follow-up. I expected her to breakdown, to be in denial, or to question me as to why her daughter had developed this disease. She did ask me some questions but remained fairly composed and requested that I give her a day to put her house in order, arrange some money, and then return to admit her daughter for initiating treatment of diabetes. I urged her to return the next day as diabetes, when untreated, can lead to catastrophic consequences in children.

As promised, Ms. S and her mother came the next morning, prepared for a hospital stay of 4–5 days to learn about diabetes, insulin injection techniques, diet management, and other nitty-gritties. Prior to ward admission, I talked to Sharika and her mother once again, briefed them about the disease and its management. Sharika was quite comfortable around me by this point and I told her that soon she will be brave enough to take the injections on her own. Her mother, who had put up a resolute front till now, broke down and told me that she had not slept a bit the previous night; she had even contemplated taking her life along with that of her child’s, as she found the burden too heavy to carry. During our conversation the previous day, she had mentioned that her husband was temporarily unable to work as he had developed disability after an accident. She wondered aloud if God had let her down and expressed her fears about how she was going to manage her daughter’s illness.

Sharika’s sugar levels had risen further and her urine tested positive for acetone, indicating a moderate degree of sickness. Thankfully, she did not develop the extreme degree of sickness with acidosis and hence did not require ICU admission. Over the next few days, our treating team taught Sharika and her mother about the disease. As they learnt about the action of insulin and symptoms associated with high or low sugar values, the mother–daughter duo became more confident about handling the disease. To my surprise, she even started taking the insulin on her own! I had hoped that she would start self-injecting eventually but the rate at which she learnt it was quite fast and I was thrilled to see it.

While they were at the hospital, her mother opened up to the senior pediatrician more about her husband’s treatment details. She had told me during the first visit that he was diagnosed with diabetes before their marriage but seemed unaware of the details of his illness. Even though it did seem a bit strange to me then, I did not prod too much, as my focus was on ensuring that they returned to the hospital for admission. On the second day of hospital stay, Sharika’s mother revealed to my senior colleague that her husband used to hide insulin vials from the family and inject himself secretly. Such was the covert nature of this insulin administration that she wondered whether he was a drug addict and was hence hiding these vials and syringes from her.

The stigma associated with insulin administration was not new to me. I had encountered adolescents who came to hospital with uncontrolled sugars as they felt awkward taking insulin to school. In the attempt to remain “normal” in front of their peers, some of them skipped insulin altogether. However, listening to the details of how a grown man had to hide his illness and medicines from his wife, hence almost causing a schism in their marital life, revealed a whole new level of stigma to me. I wondered how things might have turned out if Sharika’s father had not felt like an outcast and did not have to hide diabetes from his family. It might have helped her family to understand the illness better when Sharika was diagnosed. They might have even found it a familiar disease, one which they were already living with. But alas, it was not just the disease which had traveled to the next generation; stigma had hopped on too.

Recently, we celebrated the centenary of insulin discovery by Banting and Best. May God bless their souls, for if it was not for this simple but wonderful drug, many with diabetes would have continued to die without any treatment. That being said, insulin access still remains a huge barrier for many who will benefit from it. Stigma too remains a stumbling block in providing the best possible care to kids with diabetes. Many children struggle with their disease and treatment, especially when they arrive at the threshold of adolescence.

I hope there will be better days, when kids with diabetes do not feel like outliers and can take their insulin without having to hide it. Yesterday, I saw Sharika leaving the outpatient department after her review visit. This time she seemed to be at home, returning to a familiar spot, with a bright smile adorning her beautiful face. She told me that her sugar levels were well within the acceptable limits and her insulin dosage had been reduced. It was so good to see her, bustling with life and confidence. I pray that she will never let the world take it away from her.

